# The Subtle Pattern of Sinoatrial Wenckebach

**DOI:** 10.1016/j.jaccas.2025.106348

**Published:** 2025-12-02

**Authors:** Laith Alomari, Olayinka Adebolu, Abiodun Idowu, Ola Khraisha

**Affiliations:** Jefferson Einstein Hospital, Philadelphia, Pennsylvania, USA

**Keywords:** bradycardia, electrocardiogram, electrophysiology, sinoatrial wenckebach

## Abstract

**Introduction:**

Second-degree sinoatrial (SA) exit block type I, or SA Wenckebach, is a rare arrhythmia marked by progressively shortening P–P intervals followed by a dropped P wave and a noncompensatory pause, producing a grouped beating pattern on electrocardiogram (ECG).

**Case Presentation:**

A 64-year-old male with multiple comorbidities presented after a mechanical fall with bradycardia. ECG revealed a new SA exit block type I. Echocardiography showed preserved ejection fraction and grade II diastolic dysfunction.

**Discussion:**

Nodal blockers were discontinued, and pacemaker implantation was deemed unnecessary. Recognizing SA exit block is important, as it can mimic atrioventricular block or sinus arrhythmia on ECG.

## Graphical Abstract

**Figure:**
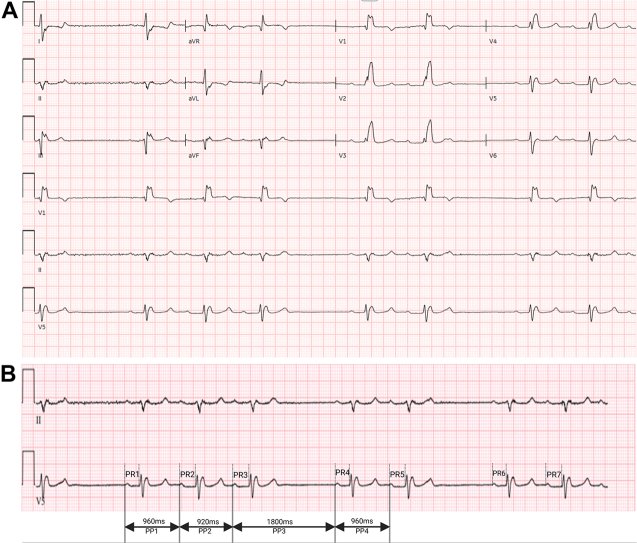

## History of Presentation

**Figure 1:**
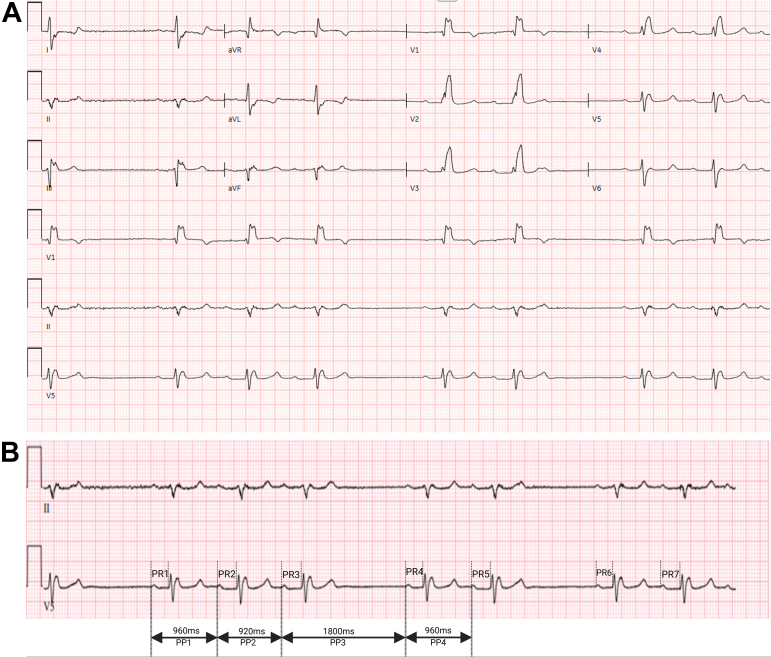
ECG on Presentation (A) ECG on presentation. (B) Rhythm strip showing progressively shortening P–P intervals, followed by a dropped P-wave and a resulting noncompensatory pause.

A 64-year-old male presented to the emergency department following a mechanical fall. His medical history included type 2 diabetes mellitus, end-stage renal disease status post kidney transplant, coronary artery disease with prior coronary artery bypass grafting (left internal mammary artery–left anterior descending artery, saphenous vein graft–first obtuse marginal artery, saphenous vein graft–posterior descending artery), heart failure with preserved ejection fraction, chronic right bundle branch block, and first-degree atrioventricular (AV) block. On examination, his heart rate was 45 beats/minute, blood pressure 150/90 mm Hg, and oxygen saturation 100% on room air. The remainder of the physical exam was unremarkable. An electrocardiogram (ECG) was obtained (Figure 1).

**Take-Home Messages**

• Second-degree sinoatrial exit block type I presents with progressively shortening P–P intervals, a dropped P-wave, and a noncompensatory pause.
• It may mimic atrioventricular block or sinus arrhythmia; accurate diagnosis requires close attention to P–P interval.
• Conservative management is appropriate for asymptomatic patients, with medication review and electrophysiology follow-up.

Current medications included metoprolol tartrate 50 mg twice daily, atorvastatin, ezetimibe, tacrolimus, prednisone, mycophenolate mofetil, patiromer, and insulin. Laboratory results showed hemoglobin 13.3 g/dL, potassium 5.2 mmol/L, creatinine 1.8 mg/dL, blood urea nitrogen 48 mg/dL, thyroid-stimulating hormone 2 mIU/L, high-sensitivity troponin I 128 ng/dL, and brain natriuretic peptide 388.8 pg/mL.

## Question

In addition to the baseline right bundle branch block, what is the most likely cardiac rhythm on the ECG?

A. Second-degree atrioventricular block, Mobitz type I
B. Second-degree atrioventricular block, Mobitz type II
C. Second-degree sinoatrial exit block type I
D. Second-degree sinoatrial exit block type II

## Discussion and Rationale

Choice A is incorrect. Mobitz type I AV block shows progressive PR interval prolongation before a dropped QRS complex. In this ECG, the PR intervals after the pause (PR1, PR4, and PR6) are 242 ms, while the other PR intervals (PR2, PR3, PR5, and PR7) are 272 ms. However, dropped P wave (not a QRS complex), grouped beating pattern, progressive PP interval shortening, and noncompensatory pause are inconsistent with AV Wenckebach.

Choice B is incorrect. Mobitz type II AV block presents with unexpected QRS drops after normal P waves. This ECG shows dropped P waves, indicating failure of impulse exit from the sinoatrial (SA) node.

Choice C is correct. The ECG displays a grouped P–QRS pattern, progressively shortening P–P intervals, and a dropped P-wave, followed by a noncompensatory pause (pause following the dropped P wave is not a multiple of the basic P–P interval). The postpause P–P interval is longer than the preceding one, consistent with second-degree SA exit block type I (SA Wenckebach).

Choice D is incorrect. SA exit block type II features fixed P–P intervals with sudden, dropped P waves. The pause is typically an exact multiple of the basic P–P interval, which is not observed here.

The SA node contains pacemaker (P) cells and transitional (T) cells. SA exit block occurs when impulses generated by P cells fail to exit through T cells.^1^ There are 3 degrees of SA exit block. First-degree SA block is not detectable on surface ECG. Second-degree SA block type I (SA Wenckebach): conduction delay progressively increases until an impulse fails to exit, producing the classic grouped beating. Second-degree SA block type II: impulses fail intermittently without prior P–P interval changes. Third-degree SA block results in complete conduction failure and is indistinguishable from sinus arrest on ECG. SA Wenckebach also contrasts with sinus arrhythmia, which features irregular but nonprogressive P–P variability and no dropped P waves.

Clinically, second-degree SA exit block type I is often benign and asymptomatic. In such cases, pacemaker implantation is not indicated. Management should focus on identifying reversible contributors, such as beta-blocker use, and arranging electrophysiology follow-up.^2^

## Funding Support and Author Disclosures

Publication made possible in part by support from the Thomas Jefferson University Open Access Fund. The authors have reported that they have no relationships relevant to the contents of this paper to disclose.

## References

[1] Manoj P., Kim J.A., Kim S. (2023). Sinus node dysfunction: current understanding and future directions. Am J Physiol Heart Circ Physiol.

[2] Petzl A.M., Epstein A.E., Guandalini G.S. (2024). Less obvious than one might think: why is there grouped beating?. Circulation.

